# Evidence for waning of latency in a cohort study of tuberculosis

**DOI:** 10.1186/1471-2334-10-37

**Published:** 2010-02-23

**Authors:** Harald G Wiker, Tehmina Mustafa, Gunnar A Bjune, Morten Harboe

**Affiliations:** 1 Section for Microbiology and Immunology, The Gade Institute, University of Bergen, Bergen, Norway; 2 Department of Microbiology and Immunology, Haukeland University Hospital, Bergen, Norway; 3 Section for International Health, Institute for General Practice and Community Medicine, University of Oslo, Oslo, Norway; 4 Institute of Immunology, University of Oslo and Oslo University Hospital, Rikshospitalet, Oslo, Norway

## Abstract

**Background:**

To investigate how the risk of active tuberculosis disease is influenced by time since original infection and to determine whether the risk of reactivation of tuberculosis increases or decreases with age.

**Methods:**

Cohort analysis of data for the separate ten year birth cohorts of 1876-1885 to 1959-1968 obtained from Statistics Norway and the National Tuberculosis Registry. These data were used to calculate the rates and the changes in the rates of bacillary (or active) tuberculosis. Data on bacillary tuberculosis for adult (20+) age groups were obtained from the National Tuberculosis Registry and Statistics Norway from 1946 to 1974. Most cases during this period arose due to reactivation of remote infection. Participants in this part of the analysis were all reported active tuberculosis cases in Norway from 1946 to 1974 as recorded in the National Tuberculosis Registry.

**Results:**

Tuberculosis decreased at a relatively steady rate when following individual birth cohorts, but with a tendency of slower decline as time passed since infection. A mean estimate of this rate of decline was 57% in a 10 year period.

**Conclusions:**

The risk of reactivation of latent tuberculosis decreases with age. This decline may reflect the rate at which latent tuberculosis is eliminated from a population with minimal transmission of tubercle bacilli. A model for risk of developing active tuberculosis as a function of time since infection shows that the rate at which tuberculosis can be eliminated from a society can be quite substantial if new infections are effectively prevented. The findings clearly indicate that preventative measures against transmission of tuberculosis will be the most effective. These results also suggest that the total population harbouring live tubercle bacilli and consequently the future projection for increased incidence of tuberculosis in the world is probably overestimated.

## Background

Depending on the epidemiological situation and the local infectious pressure, the probability of development of clinical disease after infection with *Mycobacterium tuberculosis *varies. It is often stated that among those infected, about 10% progress to active disease over their liftetime [1]. The risk of disease development is highest within the first year, estimated then to be 1%, and considerably lower the second year (0.3%) after which the risk is further gradually reduced [2]. The risk of late-appearing clinical disease is mainly due to the ability of *M. tuberculosis *to enter into a dormant stage followed by reactivation. After infection, latency can be defined as a state with no signs or symptoms of active tuberculosis. Skin test reactivity determined by intracutaneous injection of purified protein derivative (PPD) of *M. tuberculosis *and/or x-ray demonstration of a healed, calcified lesion have generally been used as evidence of previous infection or primary tuberculosis disease [3], while interferon-gamma release assays represent an important technical development for diagnosis of latent tuberculosis [4]. Observations on latent tuberculosis with *M. tuberculosis *residing in healthy individuals were made already by Opie and Aronson who cultured *M. tuberculosis *from apparently normal tissues of individuals who died from other causes, and who had no pathological evidence of tuberculosis disease [5]. During latency the bacilli show a characteristic location outside of the primary complex, i.e. in the upper lobes corresponding to the main location of cavernae in reactivation tuberculosis [6]. These findings have been extended with molecular methods [7]. Polymerase chain reaction for the IS*6110 *sequence revealed *M. tuberculosis *DNA in paraffin sections of macroscopically normal upper lobe lung tissue from 15 of 47 Ethiopian and Mexican individuals dying from causes other than tuberculosis. There are also observations indicating that the same bacterial strain induced active disease more than 20 years after original infection. From Denmark particularly interesting observations were made by comparing clinical isolates from the 1960s with isolates from the 1990s [8,9]. Two *M. tuberculosis *strains isolated from a father in 1961 and his son in 1994 had identical IS*6110 *restriction fragment length polymorphism (RFLP) pattern. The father had smear positive pulmonary tuberculosis with a one year case history in 1961, successfully treated for 12 months with streptomycin, isoniazid and para-aminosalicylic acid. His son was then 7 years old, left the home in 1972 without further contact with his father, and presented with pulmonary tuberculosis in 1994. This identical RFLP pattern of the two isolates was not found in any other strain, either among 130 historical strains collected 1961-1967 or among 4008 recent strains collected 1992-1999 [8].

An important question is for how long the latent stage of tuberculosis is continuing with a risk of reactivation and development of clinical disease. It is widely assumed that latency is life-long and that most new cases of active tuberculosis in low-endemic countries arise from a growing proportion of latently infected individuals [10], and that infected individuals thus face an increasing risk of reactivation as they grow older [11]. It is, however, possible that infected individuals gradually tend to clear the latent infection. If this is the case one may predict that the rate of reactivated tuberculosis decreases as a function of time since primary infection.

When tuberculosis came under control in Norway after the Second World War, the annual incidence of new infections diminished rapidly, and the majority of clinical cases now occurred after reactivation of remote infection [12,13]. Data from molecular epidemiological characterization of isolated *M. tuberculosis *strains in low incidence countries show that there is very little recent transmission of tuberculosis in the native population under these conditions [14-16]. Molecular fingerprinting data from Norway shows a very diverse strain repertoire with very little clustering of cases among native Norwegians. This is as expected when reactivation occurs sporadically.

The National Tuberculosis Registry and Statistics Norway provide data for new cases of bacillary tuberculosis in 10 year cohorts from the age of 20. Data are given as incidence, i.e. cases per 100,000 population per year, making it possible to follow the rate of tuberculosis in birth cohorts from young adulthood until old age. Analysis of tuberculosis in sequential birth cohorts was originally introduced by Kristian Andvord studying tuberculosis mortality at the end of the 19^th ^and beginning of the 20^th ^century [17-19]. Our analysis aimed to determine whether these data from the Norwegian experience after the Second World War can be used to estimate the duration of the latent stage and the associated risk of reactivation and development of clinical disease.

## Methods

### Setting

Data on all registered cases in Norway of bacillary tuberculosis for age specific groups in the years of 1946-1974 and for the separate ten year birth cohorts of 1876-1885 to 1959-1968 were obtained from the National Tuberculosis Registry and from Statistics Norway (Statistisk sentralbyrå) at http://www.ssb.no/aarbok/.

### Diagnosis of tuberculosis

The term "bacillary tuberculosis" was used for notification and registration of tuberculosis disease, i.e. cases being sputum smear positive, and/or culture positive for *M. tuberculosis*, and/or having cavernous tuberculosis by X-ray examination, and/or "phitisis progrediens". This definition was consistently used in Norway throughout the study period. The data in these registries are quite unique in accessing information of a total population over a very long period of time from a material that is well recognized for its high quality. A common national requirement for reporting of tuberculosis disease was important to ensure completeness of these data.

### Tuberculin testing

Extensive data on tuberculin skin testing are also accessible in the National Tuberculosis Registry. The decline of tuberculosis was analysed during the years of 1946-1974, i.e. prior to immigration affecting the tuberculosis epidemiology in Norway to avoid immigration as confounding factor in the analysis.

### Strobe analysis

STROBE analysis was performed according to the checklist of items that should be included in reports of observational studies.

### Statistical analysis

The statistical analysis was performed using SPSS version 15.0 (SPSS Inc., Chicago, Illinois; U.S.A). The significance level for all statistical tests was P < 0.05. The lowest probability recorded by the program is P < 0.0005.

## Results

The data in Table 1 show how the epidemiology of tuberculosis differed in successive birth cohorts as per January 1^st ^1973. The data are taken from figure 2 (males) and figure 3 (females) in Haider and Tverdal's publication [20]. There was a substantial decrease in percentages of spontaneous tuberculin positive individuals as well as individuals with positive X-ray findings. BCG vaccination coverage increased substantially and reached 92% in the youngest birth cohort.

**Table 1:** Background information about the cohorts per January 1^st ^1973.

Men	**Spontaneous tuberculin negative**^**1**^	**BCG vaccinated**^**2**^	**Spontaneous tuberculin positive**^**3**^	**Positive X-ray findings and/or previous tuberculosis disease**^**4**^
Birth cohort				

1949-1958	3^5^	92	3	2

1939-1948	2	86	8	4

1929-1938	4	61	25	10

1919-1928	9	30	44	17

1909-1918	13	10	52	25

1899-1908	16	1	51	32

**Women**				

Birth cohort				

1949-1958	3	92	2	3

1939-1948	4	84	8	4

1929-1938	8	62	20	10

1919-1928	13	38	35	14

1909-1918	22	13	45	20

1899-1908	27	2	44	27

The data presented in this table were taken from Haider and Tverdal [20], and were included to show the status for the cohorts with respect to tuberculin reactivity, BCG vaccination and X-ray findings towards the end of the study period.

^1^Percentage of tuberculin positive persons that later turned tuberculin negative.

^2^Percentage of BCG vaccinated persons.

^3^Percentage of tuberculin negative persons that later turned tuberculin positive without the influence of BCG vaccination.

^4^Percentage of persons with evidence for previous tuberculosis.

^5^Values represent percentage of the total population of the birth cohort.

**Figure 1:**
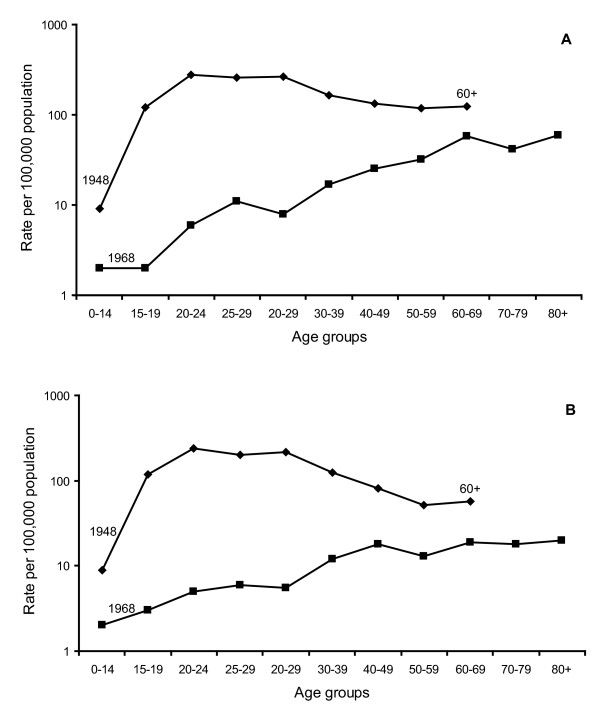
**Notified rates of bacillary tuberculosis in the various age groups of men (A) and women (B) in 1948 and 1968 respectively**. The data were obtained from the National Tuberculosis Registry and Statistics Norway.

The striking change in reporting of tuberculosis disease in Norway is illustrated in Figure 1A and 1B. In 1948 the highest rates of notified cases of active tuberculosis recorded as "bacillary tuberculosis" were seen among the young adults. In 1968 the situation had changed completely and reported case rates showed a steady increase with age. The situation as seen in 1968 is typical of a low prevalence setting and is sometimes misinterpreted to indicate that an infected person's chance of developing tuberculosis from latent infection increases in old age.

**Figure 2:**
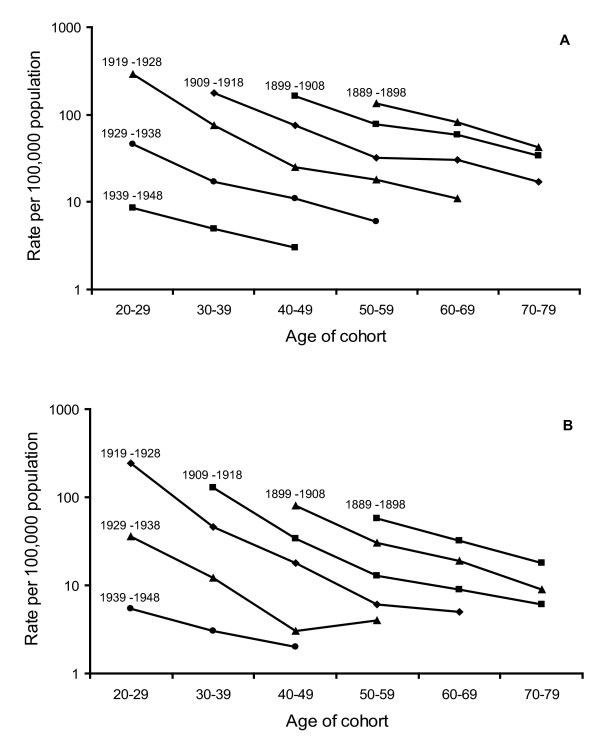
**Notified bacillary tuberculosis among 10 year cohorts of men (A) and women (B) according to age of cohort**. The data cover birth cohorts from 1889-1898 up to 1939-1948. The individual curves are labelled with the birth years of the cohorts. The data were obtained from the National Tuberculosis Registry and Statistics Norway.

The national statistics of reported tuberculosis cases in Norway provide data which enabled us to study the tuberculosis case rates in separate 10 year birth cohorts. Table 2 shows the mean annual rates of tuberculosis per 100,000 inhabitants for men and women, respectively, as notified for separate ten year birth cohorts of 1879-1888 up to 1959-1968 in the years 1948, 1958, 1968, 1978 and 1988. The graphic picture of the cohorts is shown in Figure 2A and 2B for men and women respectively. Interestingly, there is a substantial decrease in notified tuberculosis cases for all birth cohorts as they grow older and the decline appears to have followed a similar rate in all cohorts as can be seen from the largely parallel curves. This indicates that the rate of decline in the cohorts is similar in this population.

**Table 2:** Notifed cases of bacillary tuberculosis in Norway in separate birth cohorts.

	Age group					
**Men**	**20-29**	**30-39**	**40-49**	**50-59**	**60-69**	**70-79**

Birth cohort						

1959-1968	6					

1949-1958	2.5	3				

1939-1948	8.5	5	3			

1929-1938	46^2^	17	11	6		

1919-1928	286^1^	75^2^	25	18	11	

1909-1918		178^1^	76^2^	32	30	17

1899-1908			161^1^	77^2^	58	34

1889-1898				134^1^	82^2^	42

1879-1888						73^2^

**Women**	**Age group**					

Birth cohort	**20-29**	**30-39**	**40-49**	**50-59**	**60-69**	**70-79**

1959-1968	1.5					

1949-1958	2	2				

1939-1948	5.5	3	2			

1929-1938	35.5^2^	12	3	4		

1919-1928	244^1^	46^2^	18	6	5	

1909-1918		128^1^	34^2^	13	9	6

1899-1908			80^1^	30^2^	19	9

1889-1898				57^1^	32^2^	18

1879-1888						25^2^

The data were obtained from the National Tuberculosis Registry and Statistics Norway, and are sorted according to sex and age per 100,000 population in 10 year cohorts. Values represent annual rates of tuberculosis per 100,000 population. By combining the data from the birth cohort and age group it appears that the data are derived from the years 1948^1^, 1958^2^, and similarly from 1968, 1978 and 1988.

**Figure 3:**
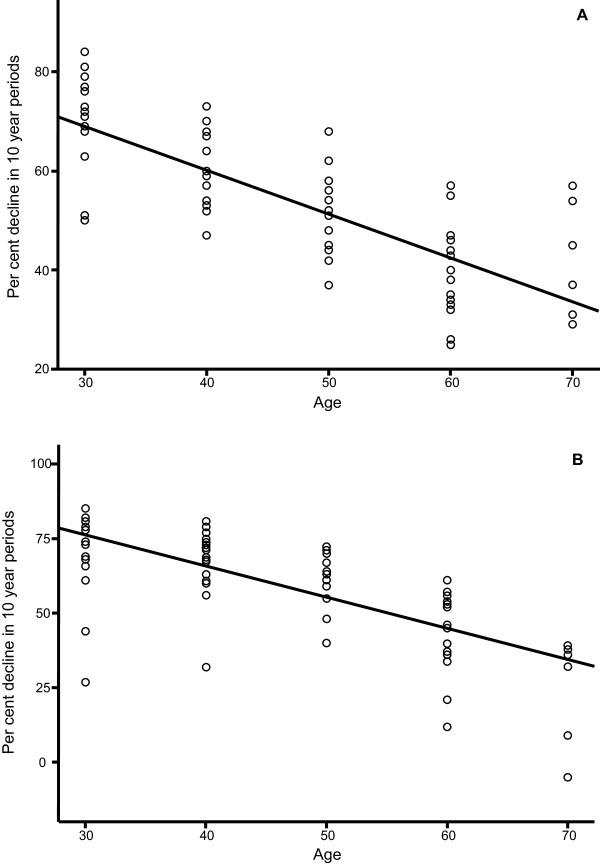
**Percentage decline over 10 year periods of bacillary tuberculosis related to mean age of 10 year cohorts of men (A) and women (B) in the period 1946 to 1974**. The observations of tuberculosis disease in 10 year cohorts were collected from 1946, 1956 and 1966; and then from another set of cohorts in 1947, 1957 and 1967 etc. The percentage change within each 10 year period was recorded as percentage decline for each cohort from 1946 to 1956 and from 1956 to 1966 etc. The figure derived from the observation of the cohort of 20-29 years in 1946 being 30-39 years in 1956 is represented as a single point in the figure under the mean age of 30 in the observation period. The figures of this age group was derived using independent observation of tuberculosis disease in the years of 1946-56, 1947-57, 1948-58 etc Each point therefore represents the observed decline within each 10 year period for every birth cohort as calculated from the rates given by the National Tuberculosis Registry and Statistics Norway. Linear curve estimation was made using the regression module of SPSS version 15.0 with the mean age of 10 year cohorts in 10 year periods as the independent variable. The curve estimations were significant with P < 0,0005 and the estimated declines were -0,882 for men (A) and -1,044 for women (B).

**Figure 4:**
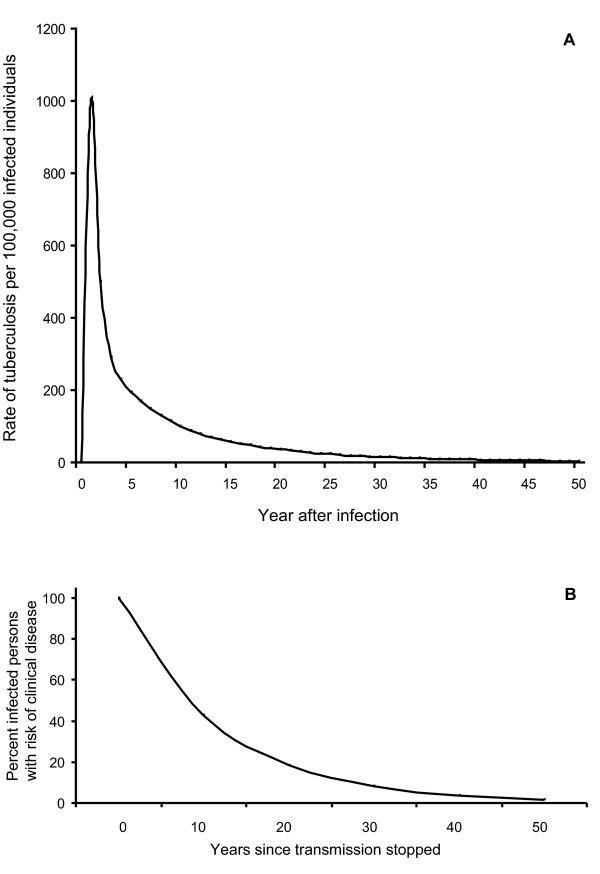
**(A) A model describing the risk of developing active tuberculosis in Norway as a function of time (in years) after infection**. The model is based on data from Ferebee [2] up to ten years after infection. The curve was extended beyond 10 years based on the expected decline in the risk of development of tuberculosis as determined by the cohort analysis of this study. **(B) This model shows the rate of clearance of latent tuberculosis from a population where all persons have been infected, and assuming that transmission of tuberculosis was completely stopped at time 0**. The cohort analysis showed an average rate of 57% decline of tuberculosis over a 10 year period, and this was used as the basis for this model. Clearance by natural death was not considered.

In order to investigate this more closely, all available data for birth cohorts from 1876 to 1954 in the period 1946 to 1974 were used to calculate the reduction in disease rates over 10 year periods. This was found to be 57% on average, 55% for men and 59% for women. The rate of decline was found to be highest for the youngest individuals, i.e. age group 20-40 years old and less marked, but still substantial for the oldest age groups (Figure 3). A similar rate of decline was found when analysing the data in relation to year of observation (data not shown). This indicates that the rate of decline is influenced by the time passed since original infection. Taken together these data were used to draw a model for risk of developing active tuberculosis as a function of time since original infection (Figure 4A). Assuming that the rate of decline of reactivated tuberculosis reflects the clearance of latent tuberculosis, a model was drawn to indicate how fast *Mycobacterium tuberculosis *can be eliminated from a population (Figure 4B).

## Discussion

Can reduction in transmission and newly infected cases explain the sharp decrease in the overall incidence of tuberculosis after 1945? This may have contributed partly in the beginning of the observation period but later there was very little transmission of tubercle bacilli. The percentages of tuberculin reactors in the cohorts with case rates above 100/100,000, i. e. prior to 1930, were quite high. Data on selected populations are available from Norway showing that among 30 year old individuals about 70% were tuberculin positive in 1927-28. This fell to 45% in 1944 and 10% in 1951-52 [21]. The birth cohorts prior to the Second World War had a substantial proportion of latently infected individuals, but removal of this fraction by death explains only a small part of the sharp decrease in notified cases. In the birth cohort of 1949-1958 only about 3-5 per cent became infected with *M. tuberculosis*. New infections had been reduced quite substantially and continued to fall in the 1960s to negligible levels. Annual conversion rates based on tuberculin testing of unvaccinated school children gives a good measure of how the level of transmission of tuberculosis in the society changed. In 1929 this was 6.4% among 2^nd ^to 6^th ^grade children [13]. This fell to 0.57% in 1950-51 and to 0.089% in 1968-69 [12]. This represents an annual decline of approximately 10% per year. The decline in transmission of tubercle bacilli was faster than the decline of bacillary cases of tuberculosis in the period, and would tend to give an overestimation of the rate of clearance of latent tuberculosis. However, considering that the majority of tuberculosis cases were due to reactivation of latent infection, the influence of recent infection for the estimation of rate of clearance of latency would be small in the 1950s and almost abolished in the 1960s and 1970s. An estimate showed that reduction in recent transmission accounts for less than 10% of the decline in total tuberculosis rates during the 1950s. Because most of the tuberculosis cases were due to remote infection the decline of tuberculosis would then reflect how latent tuberculosis became increasingly less able to establish active disease as time passed.

Any condition which can compromise host immune response would interfere with a "normal" course of waning of latent infection as studied here. This can be HIV infection or impact of war/displacement/imprisonment/malnutrition of people etc. Therefore one needs to be careful in extrapolation of these findings to other populations. Some confounding factors will have a negative influence and some factors will have a positive influence on the estimation of the decline of tuberculosis in the period. The fact that the effect is very similar in the different age groups strongly supports that the reduced incidence of tuberculosis among persons who had once been infected was due to a decreasing number of individuals harbouring live tubercle bacilli. Importantly, chemo-prophylaxis was not used in Norway, so the elimination of latent tuberculosis can not be ascribed to the use of chemotherapy.

BCG vaccination may have influenced the findings. However, BCG will mainly prevent primary tuberculosis and furthermore, BCG was given to tuberculin negative individuals only which implies that the tuberculosis infected part of the population did not receive BCG. Consequently BCG vaccination did not influence the rate of reactivation in previously infected individuals. The levels of vaccine coverage varied in the cohorts and was highest in the later cohorts (Table 1). Even so, the rate of decline of tuberculosis was highly similar between the cohorts. In conclusion, BCG vaccination had little impact on the rate of decline during the study period. This does not mean that BCG vaccination did not influence the decline of tuberculosis in Norway, only that a possible effect on reactivation was marginal at best.

Comstock [22] also reported decline of tuberculosis within birth cohorts. From his data it can be seen that the annual decrease in reported tuberculosis case rates among US adults was consistently about 5% in the birth cohorts of 1903, 1913, 1923, and 1933 when they were followed from 1953 to 1972. Similar data were obtained on the declining incidence of tuberculosis in Arkansas, USA [23], where authors used molecular fingerprinting data to estimate the relative contributions of recent and remotely acquired infection to the yearly incidence of tuberculosis. They showed that the incidence of non-clustered cases declined more dramatically than the incidence of clustered cases. This suggests that the decline in rates resulted primarily from declining rates of disease due to reactivation of past infections.

From the data given in Table 1[20], it should also be noted that a relatively high percentage of persons who had been tuberculin positive, later turned tuberculin negative. This is recorded as "spontaneous tuberculin negative" in the table and was more substantial among women than among men, an observation which is in concordance with the faster decline of bacillary tuberculosis in women (Figure 3). This conversion of a substantial proportion of tuberculin positives to tuberculin negative status is evidence for waning of the tuberculin reaction. Similar observations have been made during a long-time study on the epidemiology of tuberculosis in Ontario, Canada [24]. The tuberculin test measures delayed type hypersensitivity and depends on the presence of memory T-cells. Waning of specific delayed type hypersensitivity may indicate that mycobacterial antigen is degraded and disappear from the body. It is recently suggested that the Quantiferon responses also wane [25]. The expected infection prevalence estimated by the authors from a series of studies was 3 to 5 times higher than Quantiferon positivity rates. Longitudinal studies are however needed to confirm these observations.

In a cohort study from Hong Kong, England and Wales a similar decline of tuberculosis in cohorts late after infection was observed in England and Wales, but not in Hong Kong [26]. The authors of this paper did not reach a decisive conclusion. Our interpretation is that there was a change in the general risk of contracting tuberculosis in the Hong Kong society which appeared after 1982. This is particularly apparent in the curves for male cohorts where a change to higher tuberculosis rates kicked in simultaneously for all cohorts older than 30 years. This change is coincident with an increasing integration with the Chinese society upon the treaty between the United Kingdom and the People's Republic of China signed in 1984, and is probably due to immigration of populations from settings with a higher prevalence of tuberculosis combined with higher rates of transmission of tuberculosis in the Hong Kong society.

In conclusion, our study shows that the rate at which tuberculosis can be eliminated from a society is substantial if new infections are effectively prevented. Another conclusion is that the total population harbouring live tubercle bacilli in the world is probably overestimated, and needs to be recalculated based on rates of clearance of the latent infection. In societies with a high infection pressure such as in South Africa [27-29], high tuberculosis rates will be maintained because there will be substantial levels of exogenous re-infection contributing to maintaining latency. In societies where ongoing transmission has been reduced to a low level, the half-life of latent tuberculosis would be about 8.8 years, i.e. after 8.8 years the number of individuals with latent tuberculosis would be reduced with 50%.

## Conclusions

The current findings and analysis underscore that tuberculosis control should be targeted at transmission of infection. It is necessary to invest more to trace and treat patients with smear positive tuberculosis with minimal diagnostic delay. This is essential to counteract the recent spread of hypervirulent strains of *M. tuberculosis *[30] with an increased tendency to develop multi-drug-resistant- and extensively drug-resistant tuberculosis [31].

There is a considerable focus on research on latent tuberculosis. Molecular microbiologists are intrigued by the ability of *M. tuberculosis *to survive for years in the host and the mechanisms for reactivation are of considerable immunological interest. However, in health systems research in the global perspective it is essential to prioritise work which directly targets reduction of transmission. This will include measures to secure early diagnosis and efficient treatment of active disease as well as research into new vaccines that aim at improving protective immunity against primary tuberculosis being more efficient than conventional BCG vaccination. In several populations latent tuberculosis appears to be more rapidly cleared than previously thought.

## Competing interests

The authors declare that they have no competing interests.

## Authors' contributions

HGW contributed in study design, data collection, data analysis, data interpretation and writing of the manuscript, TM, GAB and MH participated in data analysis, data interpretation and writing of the manuscript. All authors have read and approved the final manuscript.

## Pre-publication history

The pre-publication history for this paper can be accessed here:

http://www.biomedcentral.com/1471-2334/10/37/prepub

## References

[B1] Sterling TR New approaches to the treatment of latent tuberculosis Semin Respir Crit Care Med 2008 29 532 541 10.1055/s-0028-1085704 18810686

[B2] Ferebee SH Controlled chemoprophylaxis trials in tuberculosis. A general review Bibl Tuberc 1970 26 28 106 4903501

[B3] Parrish NM Dick JD Bishai WR Mechanisms of latency in *Mycobacterium tuberculosis* Trends Microbiol 1998 6 107 112 10.1016/S0966-842X(98)01216-5 9582936

[B4] Pai M O'Brien R New diagnostics for latent and active tuberculosis: state of the art and future prospects Semin Respir Crit Care Med 2008 29 560 568 10.1055/s-0028-1085707 18810689

[B5] Opie EL Aronson JD Tubercle bacilli in latent tuberculous lesions and in lung tissue without tuberculous lesions Arch Pathol Lab Med 1927 4 1 21

[B6] Balasubramanian V Wiegeshaus EH Taylor BT Smith DW Pathogenesis of tuberculosis: pathway to apical localization Tuber Lung Dis 1994 75 168 178 10.1016/0962-8479(94)90002-7 7919306

[B7] Hernandez-Pando R Jeyanathan M Mengistu G Aguilar D Orozco H Harboe M Rook GAW Bjune G Persistence of DNA from *Mycobacterium tuberculosis *in superficially normal lung tissue during latent infection Lancet 2000 356 2133 2138 10.1016/S0140-6736(00)03493-0 11191539

[B8] Lillebaek T Dirksen A Baess I Strunge B Thomsen VØ Molecular evidence of endogenous reactivation of *Mycobacterium tuberculosis *after 33 years of latent infection J Infect Dis 2002 185 401 404 10.1086/338342 11807725

[B9] Lillebaek T Dirksen A Vynnycky E Baess I Thomsen VØ Andersen AB Stability of DNA patterns and evidence of *Mycobacterium tuberculosis *reactivation occurring decades after the initial infection J Infect Dis 2003 188 1032 1039 10.1086/378240 14513424

[B10] Lin MY Ottenhoff TH Host-pathogen interactions in latent *Mycobacterium tuberculosis *infection: identification of new targets for tuberculosis intervention Endocr Metab Immune Disord Drug Targets 2008 8 15 29 10.2174/187153008783928398 18393920

[B11] Borgdorff MW Werf MJ van der de Haas PE Kremer K van Soolingen D Tuberculosis elimination in the Netherlands Emerg Infect Dis 2005 11 597 602 15829200 10.3201/eid1104.041103 PMC3320334

[B12] Bjartveit K [Article in Norwegian][Situation of tuberculosis in Norway] Tidsskr Nor Laegeforen 1975 95 1049 1055 1145585

[B13] Dahl E Backer J [Article in Norwegian][Tuberculosis in Oslo during the war] Nor Mag Laegevid 1945 108 2305 2313

[B14] Dahle UR Sandven P Heldal E Caugant DA Continued low rates of transmission of *Mycobacterium tuberculosis *in Norway J Clin Microbiol 2003 41 2968 2973 10.1128/JCM.41.7.2968-2973.2003 12843028 PMC165220

[B15] Dahle UR Sandven P Heldal E Caugant DA Molecular epidemiology of *Mycobacterium tuberculosis *in Norway J Clin Microbiol 2001 39 1802 1807 10.1128/JCM.39.5.1802-1807.2001 11325994 PMC88029

[B16] Bandera A Gori A Catozzi L Degli EA Marchetti G Molteni C Ferrario G Codecasa L Penati V Mateelli A Franzetti F Molecular epidemiology study of exogenous reinfection in an area with a low incidence of tuberculosis J Clin Microbiol 2001 39 2213 2218 10.1128/JCM.39.6.2213-2218.2001 11376059 PMC88113

[B17] Andvord KF [Original article in Norwegian][What can we learn by following the development of tuberculosis from one generation to another?] Nor Mag Laegevid 1930 91 642 660

[B18] Andvord KF [Translation of original article published in 1930][What can we learn by following the development of tuberculosis from one generation to another?] Int J Tuberc Lung Dis 2002 6 562 568 12102293

[B19] Blomberg B Rieder HL Enarson DA Kristian Andvord's impact on the understanding of tuberculosis epidemiology Int J Tuberc Lung Dis 2002 6 557 559 12102291

[B20] Haider T Tverdal A [Article in Norwegian][Prevalence of tuberculosis infections] Tidsskr Nor Laegeforen 1982 102 1065 1068 7164059

[B21] Sandberg HK [Article in Norwegian][Register of tuberculin test results and decrease in tuberculosis; indications for continuing present control procedures] Tidsskr Nor Laegeforen 1954 74 317 322 13169118

[B22] Comstock GW Frost revisited: the modern epidemiology of tuberculosis Am J Epidemiol 1975 101 363 382 1093397 10.1093/oxfordjournals.aje.a112105

[B23] France AM Cave MD Bates JH Foxman B Chu T Yana Z What's driving the decline in tuberculosis in Arkansas? A molecular epidemiologic analysis of tuberculosis trends in a rural, low-incidence population, 1997-2003 Am J Epidemiol 2007 166 662 671 10.1093/aje/kwm135 17625223

[B24] Grzybowski S Allen EA The challenge of tuberculosis in decline. A study on the epidemiology of tuberculosis in Ontario, Canada Am Rev Respir Dis 1964 90 707 720 14211457 10.1164/arrd.1964.90.5.707

[B25] Mori T Harada N Higuchi K Sekiya Y Uchimura K Shimao T Waning of the specific interferon-gamma response after years of tuberculosis infection Int J Tuberc Lung Dis 2007 11 1021 1025 17705982

[B26] Tocque K Bellis MA Tam CM Chan SL Syed Q Remmington T Davies PD Long-term trends in tuberculosis. Comparison of age-cohort data between Hong Kong and England and Wales Am J Respir Crit Care Med 1998 158 484 488 9700125 10.1164/ajrccm.158.2.9709125

[B27] Fine PE Small PM Exogenous reinfection in tuberculosis N Engl J Med 1999 341 1226 1227 10.1056/NEJM199910143411609 10519901

[B28] van Rie A Warren R Richardson M Victor TC Gie RP Enarson DA Beyers N van Elden PD Exogenous reinfection as a cause of recurrent tuberculosis after curative treatment N Engl J Med 1999 341 1174 1179 10.1056/NEJM199910143411602 10519895

[B29] Warren RM Victor TC Streicher EM Richardson M Beyers N Gey van Pittius NC van Elden PD Patients with active tuberculosis often have different strains in the same sputum specimen Am J Respir Crit Care Med 2004 169 610 614 10.1164/rccm.200305-714OC 14701710

[B30] Victor TC Streicher EM Kewley C Jordaan AM Spuy GD van der Bosman M Louw H Murray M Young D van Helden PD Warren RM Spread of an emerging *Mycobacterium tuberculosis *drug-resistant strain in the western Cape of South Africa Int J Tuberc Lung Dis 2007 11 195 201 17263291

[B31] Mlambo CK Warren RM Poswa X Victor TC Duse AG Marais E Genotypic diversity of extensively drug-resistant tuberculosis (XDR-TB) in South Africa Int J Tuberc Lung Dis 2008 12 99 104 18173885

